# Burkitt-Type Acute Lymphoblastic Leukemia With Precursor B-Cell Immunophenotype and Partial Tetrasomy of 1q: A Case Report

**DOI:** 10.1097/MD.0000000000002904

**Published:** 2016-03-11

**Authors:** Yuya Sato, Hidemitsu Kurosawa, Keitaro Fukushima, Mayuko Okuya, Osamu Arisaka

**Affiliations:** From the Department of Pediatrics, Dokkyo Medical University School of Medicine, Mibu, Tochigi, Japan.

## Abstract

Burkitt-type acute lymphoblastic leukemia (B-ALL) is thought as a variant of Burkitt lymphoma/leukemia and derived from mature B-cell lymphoblast.

B-ALL was developed in a 10-year-old girl. Two characteristics were apparent in this case. First, the lymphoblastic cells were positive for CD10, CD19, CD20, and CD22, but negative for terminal deoxynucleotidyl transferase and surface immunoglobulins, indicating a B-cell immunophenotype. The detection of t(8;14)(q24;q32) with a chromosomal analysis is required for a diagnosis of B-ALL. Second, der(1)(pter → q32.1::q32.1 → q21.1::q11 → qter) was detected, in which 1q21.1 to 1q32.1 was inverted and inserted. Finally, partial tetrasomy of 1q was also present. Because B-ALL with abnormal chromosome 1 has been reported poor outcome, the usual chemotherapy for stage 4 Burkitt lymphoma with added rituximab was administered for our patient.

We report B-ALL with precursor B-cell immunophenotype and interesting partial tetrasomy of 1q.

## INTRODUCTION

Burkitt-type acute lymphoblastic leukemia (B-ALL) is classified as a variant of Burkitt lymphoma/leukemia. A combination of several diagnostic methods, including morphological, cytogenetic, and chromosomal analyses and immunophenotyping, is necessary to diagnose B-ALL with certainty.^1,2^ B-ALL presents with a French–American–British (FAB) classification L3 morphology and a mature B-cell immunophenotype, with the surface expression of CD20 and surface immunoglobulins with clonally restricted light chains.^1,2^ According to the 2008 World Health Organization (WHO) classification, a translocation involving *MYC*, t(8;14)(q24;q32), is highly characteristic of B-ALL, resulting in the constitutive expression of the *MYC* gene.^2,3^

In a study by the Pediatric Oncology Group, B-ALL with a precursor B-cell immunophenotype was shown to occur in approximately 0.1% of children with ALL.^4–6^ Additional chromosomal aberrations can be present, with chromosomes 1, 6, 7, 13, 17, and 22 most commonly affected.^2,7^ We describe a case of B-ALL with a precursor B-cell immunophenotype and partial tetrasomy of 1q.

### Case Report

**FIGURE 1:**
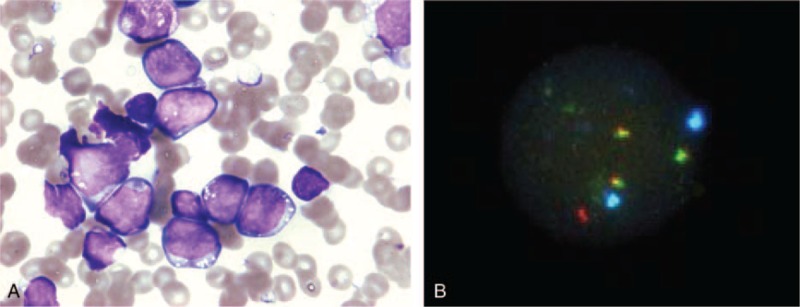
May–Giemsa staining and fluorescent in situ hybridization analysis. (A) May–Giemsa-staining-based morphological analysis revealed many cytoplasmic vacuoles but inconspicuous granules. The blasts were not typical of the FAB classification of L3. (B) Fluorescent in situ hybridization analysis using an *IgH/C-MYC* probe. The dual fusion translocation probe hybridized to the nucleus. The 3 yellow spots are *IGH/C-MYC* fusion gene signals.

**FIGURE 2:**
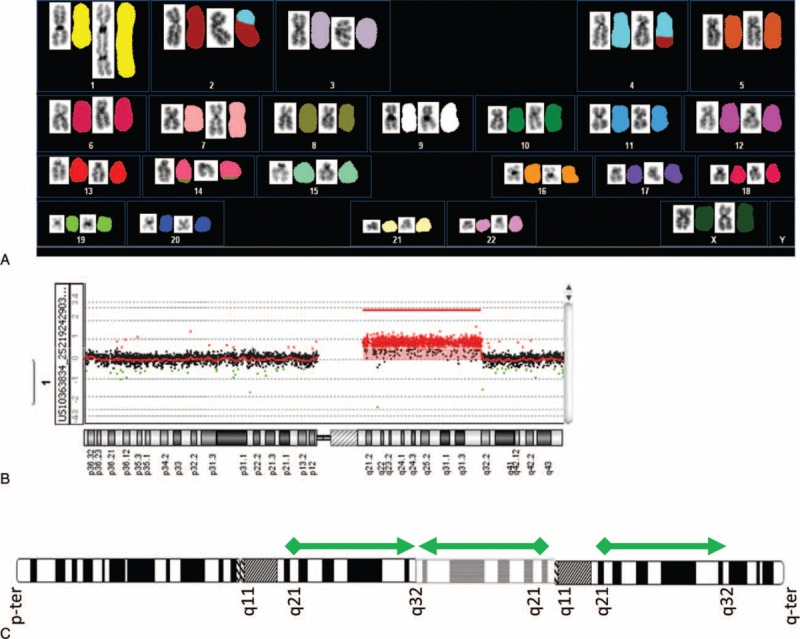
G-band-staining-based chromosome analysis, spectral karyotyping fluorescent in situ hybridization (SKY-FISH) analysis, and array-based comparative genomic hybridization (aCGH) array. (A) G-band-stained chromosomes and SKY-FISH suggested 46XY and an additional abnormal karyotype involving t(8;14)(q24;q32), t(2;4)(p13;q27), and der(1)(pter → q32.1::q32.1 → q21.1::q11 → qter). (B) aCGH array of chromosome 1 revealed partial gain of 1q21.1 to 1q32.1. This region conferred the partial tetrasomy of 1q. (C) Schema of chromosome 1.

A 10-year-old girl was admitted to our hospital complaining of lassitude and fever. She displayed hepatosplenomegaly and pleural and abdominal effusions without lymphadenopathy. Lactate dehydrogenase (10,554 U/L; normal range 106–211 U/L) was elevated. A peripheral blood examination showed the following counts: red blood cells 4.25 × 10^10^/L, hemoglobin 11.8 g/dL, hematocrit 36.2%, platelets 7.3 × 10^8^/L, and white blood cells 4.4 × 10^7^/L, and 48% of leukemic blasts had vacuoles in the cytoplasm (Figure 1A). Leukemic blasts accounted for 73.6% of the mononuclear bone-marrow cells. An immunophenotype analysis was positive for CD10, CD19, CD20, and CD22, and negative for terminal deoxynucleotidyl transferase and surface immunoglobulins, suggesting that the leukemic blasts had a precursor B-cell immunophenotype. A fluorescence in situ hybridization (FISH) analysis revealed an *IgH/C-MYC* gene fusion (Figure 1B). A G-band-staining chromosomal analysis revealed t(8;14) and an additional abnormality of chromosome 1. A spectral karyotyping FISH analysis and array-based comparative genomic hybridization (aCGH) were performed. A derivative chromosome 1 analysis revealed der(1)(pter → q32.1::q32.1 → q21.1::q11 → qter), in which region 1q21.1 to 1q32.1 was inverted, inserted (Figure 2A–C), and repeated 3 times, causing partial tetrasomy of 1q (Figure 2B and C). Another abnormality, t(2;4)(p13;q27), was also detected (Figure 2A). B-ALL with a precursor B-cell immunophenotype and 46,XY,t(8;14)(q24;q32),t(2;4)(p13;q27),der(1)(pter → q32.1:: q32.1 → q21.1::q11 → qter) was diagnosed (Figure 2A–C).

Dialysis was performed for tumor lysis syndrome before induction therapy was administered. Complete remission was achieved after B-precursor ALL induction therapy with prednisolone, vincristine, and doxorubicin. The consolidation therapy was adapted from a regimen for advanced Burkitt lymphoma, according to the Japan Pediatric Leukaemia/Lymphoma Study Group B-NHL 03 advanced-stage protocol, including 6 courses of prednisolone, vincristine, cyclophosphamide, methotrexate, pirarubicin, and etoposide. Rituximab was combined with the chemotherapy in last 2 courses. This patient has been in remission for 2 years.

## DISCUSSION

B-ALL with a precursor B-cell immunophenotype has been shown to occur in approximately 0.1% of children with ALL, and a chromosomal analysis to detect t(8;14) is required for a correct diagnosis of B-ALL.^1–6^ Almost all patients suffering B-ALL with a precursor B-cell immunophenotype are administered induction therapy for B-cell ALL, and after a correct diagnosis is made, consolidation chemotherapy for B-ALL is commenced.^4,6,8,9^ It is unclear whether the progression of B-ALL with a precursor B-cell immunophenotype is worse than that of B-ALL, but because B-ALL is responds well to treatment, B-ALL with a precursor B-cell immunophenotype is expected to behave similarly.

Chromosomal aberrations in addition to t(8;14) are often present in B-ALL, most commonly affecting chromosomes 1, 6, 7, 13, 17, and 22. The most frequent additional aberration involves the long arm of chromosome 1, typically as partial tetrasomy of 1q.^2,7,10^ The partial tetrasomy of 1q observed in our patient was caused by the inversion and insertion of chromosomal region 1q21.1 to 1q32.1 between 1q32.1 and 1q11. To the best of our knowledge, B-ALL with this partial tetrasomy of 1q, der(1)(pter → q32.1:: q32.1 → q21.1::q11 → qter), has not previously been reported.

Over 60% of the patients diagnosed with B-ALL and additional aberrations of chromosome 1 relapse or die.^2,7,10^ In our patient, the partial tetrasomy of 1q arose from the inversion of chromosomal region 1q21.1 to 1q32.1. The duplication of 1q21.1 has been reported in neuroblastoma,^11^ and the development of familial nonmedullary thyroid cancer has been linked to the distribution of 1q21.1.^12^ The wide variety of lymphomas associated with an abnormality of 1q21.1 has been also reported, including Burkitt lymphoma.^2,10,13–20^ It is interesting that malignant neoplasms with 1q21.1 confer poor prognoses. These facts suggest that our patient also has a poor prognosis. However, the partial tetrasomy of 1q involved the chromosomal region from 1q21.1 to 1q32.1, which was not only inserted but also inverted in our patient. We also speculate that the inverted chromosomal region 1q21.1 to 1q32.1 may act as an antisense molecule. The antisense molecule may induce different prognosis compared with malignant neoplasms with 1q21.1.

Based on our hypothesis and the fact that B-ALL with more than 3 cytogenetic abnormalities confers a poor prognosis,^7^ rituximab was added to the consolidation therapy for B-ALL in our patient to ensure a good outcome.^21^ The patients have been in complete remission for 3 years since chemotherapy. We also think that stem-cell transplantation would be an effective intervention, but has not been available to our patient because no human leukocyte antigen (HLA)-matched donor has been found.
